# Na^+^ Transporter SvHKT1;1 from a Halophytic Turf Grass Is Specifically Upregulated by High Na^+^ Concentration and Regulates Shoot Na^+^ Concentration

**DOI:** 10.3390/ijms21176100

**Published:** 2020-08-24

**Authors:** Yuki Kawakami, Shahin Imran, Maki Katsuhara, Yuichi Tada

**Affiliations:** 1 Graduate School of Bionics, Computer and Media Sciences, Tokyo University of Technology, 1404-1 Katakura, Hachioji, Tokyo 192-0982, Japan; g11170090f@edu.teu.ac.jp; 2 Institute of Plant Science and Resources, Okayama University, Chuo 2-20-1, Kurashiki, Okayama 710-0046, Japan; ptj87a5q@s.okayama-u.ac.jp (S.I.); kmaki@okayama-u.ac.jp (M.K.); 3 School of Biosciences and Biotechnology, Tokyo University of Technology, 1404-1 Katakura, Hachioji, Tokyo 192-0982, Japan

**Keywords:** *Arabidopsis thaliana*, halophyte, high-affinity potassium transporter (HKT), Na^+^ transporter, salt tolerance, *Sporobolus virginicus*

## Abstract

We characterized an Na^+^ transporter SvHKT1;1 from a halophytic turf grass, *Sporobolus virginicus*. SvHKT1;1 mediated inward and outward Na^+^ transport in *Xenopus laevis* oocytes and did not complement K^+^ transporter-defective mutant yeast. SvHKT1;1 did not complement *athkt1;1* mutant *Arabidopsis*, suggesting its distinguishable function from other typical HKT1 transporters. The transcript was abundant in the shoots compared with the roots in *S. virginicus* and was upregulated by severe salt stress (500 mM NaCl), but not by lower stress. *SvHKT1;1*-expressing *Arabidopsis* lines showed higher shoot Na^+^ concentrations and lower salt tolerance than wild type (WT) plants under nonstress and salt stress conditions and showed higher Na^+^ uptake rate in roots at the early stage of salt treatment. These results suggested that constitutive expression of *SvHKT1;1* enhanced Na^+^ uptake in root epidermal cells, followed by increased Na^+^ transport to shoots, which led to reduced salt tolerance. However, Na^+^ concentrations in phloem sap of the SvHKT1;1 lines were higher than those in WT plants under salt stress. Based on this result, together with the induction of the *SvHKT1;1* transcription under high salinity stress, it was suggested that SvHKT1;1 plays a role in preventing excess shoot Na^+^ accumulation in *S. virginicus*.

## 1. Introduction

Soil salinity is one of the major environmental stress factors, causing significant losses in global agricultural productivity [1]. To fight this problem, it is necessary to develop salt-tolerant crops, which require a better understanding of the physiological mechanisms controlling salinity tolerance in plants. Salt stress imposes both osmotic and ionic stresses, and then oxidative stress caused by these stresses. Osmotic imbalance causes water deficit, reduced leaf area expansion, and stomatal closure, which ultimately lessen photosynthesis and growth [2]. Toxic Na^+^ can accumulate in the cytoplasm and cause imbalances in the absorption of other essential ions such as K^+^, leading to malfunction of essential biochemical and physiological processes [3]. Na^+^ has a strong inhibitory effect on K^+^ uptake by cells [4]. The increase in cytoplasmic Na^+^ and reduction of K^+^ result in changes in membrane potential, osmotic pressure, turgor pressure, calcium signaling, reactive oxygen species signaling, and transcriptional regulation, as well as alteration of gene expression and modification of protein expression pattern and spectra of small interfering RNAs (siRNAs), signaling molecules, phytohormones, and metabolites [5]. K^+^ deficiency can disrupt various enzymatic processes and impose an energetic burden on the cell owing to the requirement of organic solute synthesis to compensate for the export of Na^+^ for osmotic adjustment [1]. More than 50 enzymes are activated by K^+^, which cannot be substituted with Na^+^ [6]. Therefore, it is important to understand how Na^+^ is taken up and transported in plants under saline conditions. In order to maintain a high cytosolic K^+^/Na^+^ ratio, plants have different K^+^ and Na^+^ transporters to protect the plant against damage due to toxic Na^+^ accumulation [7,8]. To maintain low cytoplasmic Na^+^ concentrations and cell turgor pressure, Na^+^ can be sequestrated into vacuoles by the tonoplast-localized Na^+^/H^+^ exchanger 1 (NHX1) [9]. In the root epidermis cells, a plasma membrane-localized Na^+^/H^+^ antiporter (SOS1) extrudes Na^+^ back to the soil in a mechanism coupled to H^+^ transport [10]. Other Na^+^ transporters also play crucial roles in salinity tolerance by controlling the Na^+^ movement throughout the plant. Na^+^ enters roots passively, via non-selective cation channels [11] and possibly other Na^+^ transporters such as high-affinity potassium transporters (HKTs) [6]. The HKTs permeable either to Na^+^ only (HKT1) or to K^+^ and Na^+^ (HKT2) are thought to play major roles in controlling Na^+^ accumulation in plants. For a detailed overview of the physiological roles of HKTs, see the review by Almeida et al. [7]. The HKT2s were shown to have a role in Na^+^ uptake from the external medium. In rice, OsHKT2;1 catalyzes Na^+^ uptake in low K^+^, low Na^+^ (<2 mM) conditions [12]. OsHKT2;1 functions as a relatively Na^+^-specific transporter that mediates Na^+^ influx in K^+^-starved roots and, thus, promotes their growth [12,13]. Overexpression of *HvHKT2;1* in barley causes increased Na^+^ uptake in salt stress conditions [14]. Similarly, altered expression of *TaHKT2;1* in wheat affected Na^+^ accumulation in the low-affinity range [15]. We also reported that SvHKT2;1 and SvHKT2;2 from a halophyte, *Sporobolus virginicus,* mediate both K^+^ and Na^+^ transport in transgenic *Arabidopsis*, *Xenopus* *laevis* oocytes, and yeast [16]. *S. virginicus* is a halophytic C_4_ grass and shows a salinity tolerance up to 1.5 M NaCl [17]. The HKT1s play major roles in Na^+^ transport. In *Arabidopsis*, the HKT family comprises a single member, AtHKT1;1, which is permeable to Na^+^ only [18] and contributes to Na^+^ removal from the ascending xylem sap and Na^+^ recirculation from the leaves to the roots via the phloem vasculature [19,20,21,22]. Similarly, Na^+^ removal from the root xylem sap and/or shoot phloem was reported for other HKT1s including OsHKT1;1, OsHKT1;4, and OsHKT1;5 in rice [23,24,25,26,27], TaHKT1;5-D in bread wheat [28], HvHKT1;1 and HvHKT1;5 in barley [29,30], and TmHKT1;5-A in wheat [31]. On the other hand, a halophytic relative of *Arabidopsis*, *Eutrema salsuginea* (previously *Thellungiella halophila* or *Thulengiella salsuginea*), possessed three copies of *HKT1* genes [32]. Of the three, *EsHKT1;1* and *EsHKT1;2* showed high affinity for Na^+^ and K^+^, respectively, in yeast [33]. Another *Arabidopsis* halophytic relative, *Eutrema parvula* (*Schrenkiella parvula*), possesses two *HKT1* genes *EpHKT1;1* and *EpHKT1;2* [34]. Although EpHKT1;2 and EsHKT1;2 belong to HKT1, they show K^+^ uptake ability, which makes them functionally different from other members of HKT1 [35]. Thus, the ion permeability of HKT1s differs depending on the plant species. However, there is no report on the functions of HKT1s from halophytic monocotyledonous plants.

In this study, we isolated a gene for sodium transporter *SvHKT1;1* from a halophytic turf grass, *Sporobolus virginicus,* and revealed its unique expression profile, ion permeability, and possible functions in salt tolerance.

## 2. Results

### 2.1. Comparison of Amino-Acid Sequences of SvHKT1;1 and Other HKTs

**Figure 1:**
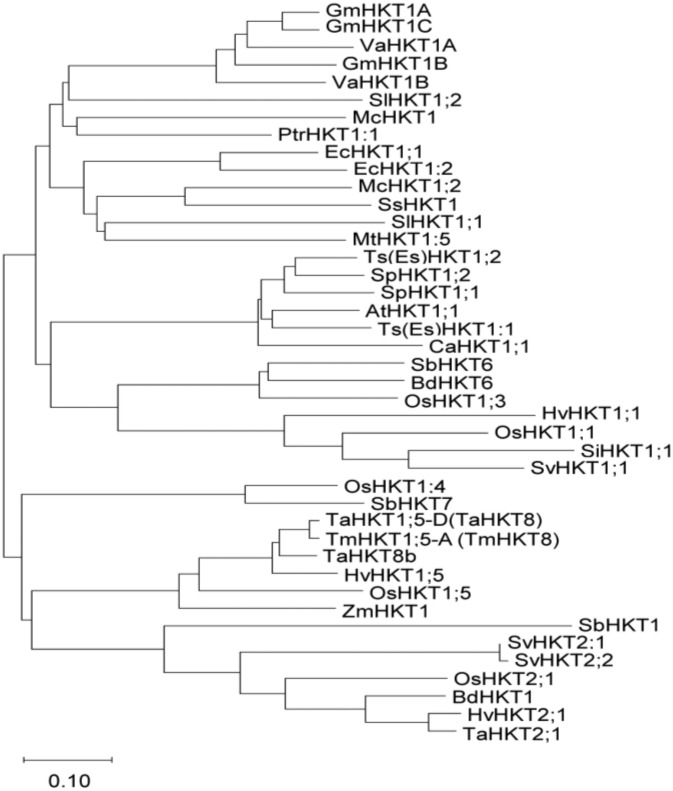
Phylogenetic analysis of high-affinity potassium transporters (HKTs). A phylogenetic analysis of the selected HKT amino-acid sequences was performed using the neighbor-joining method in the MEGA-X [39] software package. Accession numbers of amino-acid sequences used are listed in Supplementary Figure S1. The branch length is proportional to the evolutionary distance between the HKTs, indicating the number of amino-acid changes per site. The scale bar shows a length corresponding to 0.10 of the value.

Among the unigenes of *S. virginicus* [36], we searched for genes that are homologous to known *HKT* and identified *SvHKT1;1,* which belongs to class I *HKT* genes because its deduced amino-acid (AA) sequences contain a serine in the first P-loop (Supplementary Figure S1) [37,38]. Phylogenetic analysis following alignment of the AA sequences of SvHKT1;1 with other class I HKTs indicated an evolutionally close relationship to *Setaria italica* (foxtail millet) SiHKT1;1 and rice OsHKT1;1 (Figure 1).

### 2.2. Expression of SvHKT1;1 Gene and Na^+^ Concentration in S. virginicus under Salt Stress

**Figure 2:**
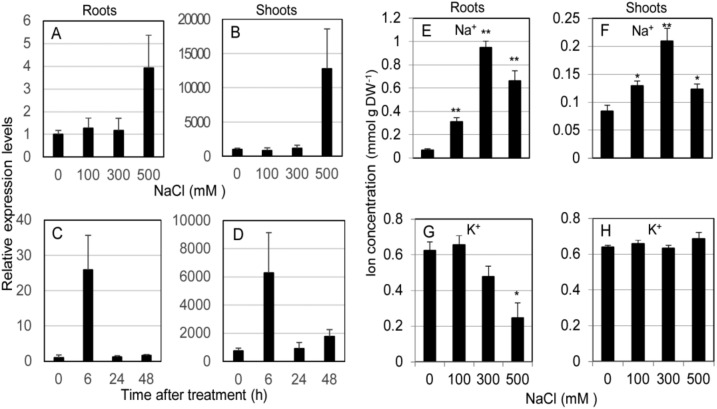
Expression profile of *SvHKT1;1* gene and Na^+^ concentrations in *Sporobolus virginicus* under salt stress. The expression profile of *SvHKT1;1* gene and Na^+^ concentrations in roots and shoots of hydroponically grown *S. virginicus* were determined. (**A**–**D**) Expression levels of *SvHKT1;1* gene determined by qRT-PCR under different NaCl concentrations (**A**,**B**) or at different time points after salt treatment (**C**,**D**). Plants grown in 1/2 Murashige and Skoog (MS) medium were transferred to 1/2 MS medium supplemented with 0, 100, 300, or 500 mM NaCl, and the roots (**A**) and shoots (**B**) were harvested at 48 h after the treatment. Plants grown in 1/2 MS medium were transferred to 1/2 MS medium supplemented with 500 mM NaCl, and the roots (**C**) and shoots (**D**) were harvested at indicated time points. Expression levels relative to that in roots at 0 h after treatment (1.0) are shown. *eIF3* was used as a reference gene. (**E**–**H**) Na^+^ (**E**,**F**) and K^+^ (**G**,**H**) concentrations in roots (**E**,**G**) and shoots (**F**,**H**) of hydroponically grown *S. virginicus* under different NaCl concentrations. The roots and shoots were harvested at 48 h after the treatment. Data are presented as means ± SE (*n* = 3 biological replicates). Single and double asterisks denote significant differences compared with the values of WT plants of the same conditions at *p* < 0.05 and *p* < 0.01, respectively, determined using the Student’s *t*-test.

The expression profile of *SvHKT1;1* gene in roots and shoots of *S. virginicus* was determined by qRT-PCR using *eIF3* as a reference gene under different salt stress conditions. *SvHKT1;1* transcript was found to preferentially accumulate in shoots compared with roots under any conditions (Figure 2A–D). In experiments at different salt concentrations, the expression stayed at similar levels under 0–300 mM NaCl conditions but significantly increased under 500 mM NaCl in both roots and shoots (Figure 2A,B). In a time-course experiment, the expression in both shoots and roots were upregulated 6 h after treatment with 500 mM NaCl, then decreased to levels several times higher than that at 0 h until 48 h after the treatment (Figure 2C,D). Similar expression profiles were observed by qRT-PCR analysis using *actin* as a reference gene (Supplementary Figure S2). Thus, *SvHKT1;1* expression is specifically upregulated by high Na^+^ concentration and preferentially detected in shoots.

Na^+^ and K^+^ concentrations in roots and shoots of *S. virginicus* were determined under different salt stress conditions. Na^+^ was found to preferentially accumulate in roots compared with shoots under salt stress (Figure 2E,F). Shoot and root Na^+^ concentration linearly increased in accordance with the NaCl concentration up to 300 mM NaCl but dropped at 500 mM, when *SvHKT1;1* transcription was dramatically upregulated (Figure 2E,F). On the other hand, root K^+^ concentration decreased with increasing external NaCl concentration but shoot K^+^ concentration remained constant (Figure 2G,H).

### 2.3. Localization of SvHKT1;1 in Nicotiana benthamiana Cells

**Figure 3:**
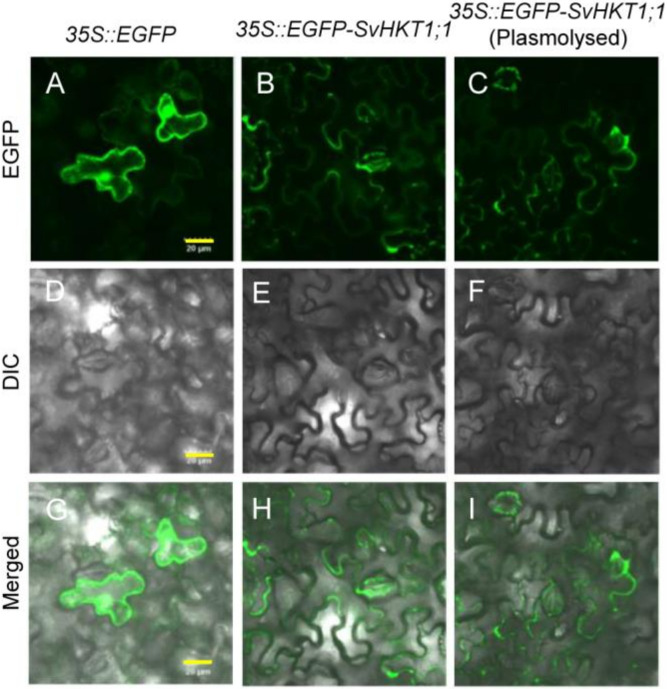
Subcellular localization of EGFP-fused SvHKT1;1 protein in *Nicotiana benthamiana* leaves. Confocal fluorescence images of EGFP (**A**–**C**), differential interference contrast images (**D**–**F**), and merged images (**G**–**I**) of *N. benthamiana* leaf cells expressing EGFP control (**A**,**D**,**G**) and EGFP–SvHKT1;1 (**B**,**C**,**E**,**F**,**H**,**I**). Images of non-plasmolyzed (**A**–**F**) and plasmolyzed (**G**–**I**) cells. Scale bar represents 20 μm and is applicable to all panels in this figure.

To investigate the intracellular localization of EGFP–SvHKT1;1 fusion protein, *Agrobacterium* expressing *EGFP–SvHKT1;1* or a control *EGFP*-only construct were infiltrated into *N. benthamiana* leaf cells, and the fluorescence signals were observed using confocal laser scanning microscopy (Figure 3). EGFP–SvHKT1;1 fusion protein specifically localized to the plasma membrane (Figure 3B,E,H). In contrast, when EGFP alone was expressed, it localized to the nucleus and the cytoplasm (Figure 3A,D,G). This pattern was verified by treatment with a hypertonic solution, 0.5 M mannitol, which induced plasmolysis (Figure 3C,F,I), indicating SvHKT1;1 localized to the plasma membrane.

### 2.4. Functional Analysis of SvHKT1;1 in X. laevis Oocytes

**Figure 4:**
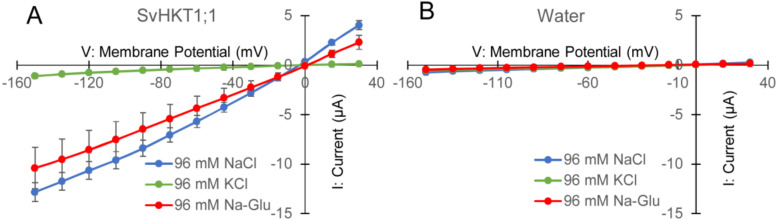
Analyses of SvHKT1;1-mediated ion transport by two electrode voltage clamp experiments using *Xenopus laevis* oocytes. Current–voltage relationship of oocytes injected with 12.5 ng of *SvHKT1;1* complementary RNA (cRNA) (**A**) or water (**B**) bathed in solutions containing an indicated amount of NaCl, KCl, or Na-gluconate. Voltage steps ranged from −150 to +30 mV with 15-mV increments. Data are presented as means ± SD (*n* = 3–7).

To examine K^+^ and/or Na^+^ transporter activities in *X. laevis* oocytes, *SvHKT1;1* complementary RNAs (cRNAs) were injected into oocytes, and the electrophysiological profile was analyzed. In two-electrode voltage clamp (TEVC) experiments, SvHKT1;1 produced inward and outward currents (Figure 4A) when oocytes were bathed in both NaCl and Na-gluconate solutions, but ion currents were hardly detected in KCl solution (Figure 4A). These results indicate that the SvHKT1;1 transporter mediates Na^+^, but not K^+^ or Cl^−^, transport. On the other hand, water-injected control oocytes showed small background currents in the same conditions (Figure 4B).

### 2.5. SvHKT1;1 Does Not Complement Yeast with Defective K^+^ Transporters

To examine K^+^ channel/transporter activities in yeast, *SvHKT1;1* was expressed in yeast strain 9.3 with defective K^+^ transporters (Supplementary Figure S3). Yeast lines harboring *SvHKT1;1* and a negative control expressing an empty vector showed similar poor growth on agar containing 0.2 mM K^+^, whereas yeast lines harboring positive control *SvHKT2;1* grew better than *SvHKT1;1* and negative control lines (Supplementary Figure S3). These results indicate that *SvHKT1;1* does not complement the K^+^-uptake deficiency in the mutant yeast.

### 2.6. SvHKT1;1 Does Not Complement athkt1;1 Mutant Arabidopsis Plants

**Figure 5:**
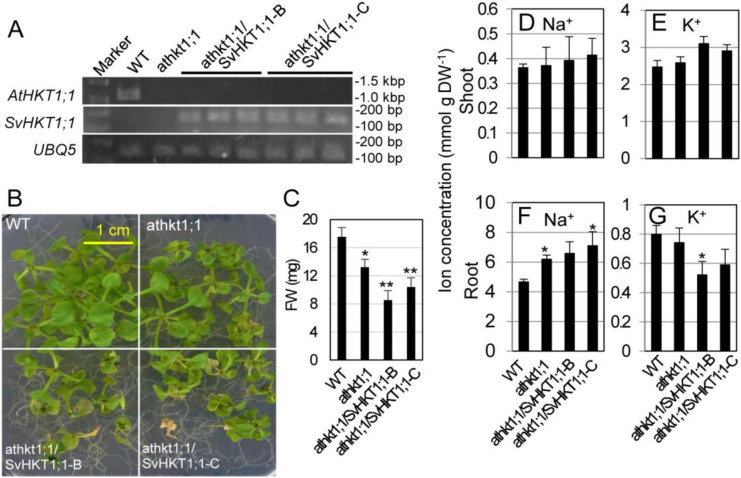
Complementation test of *athkt1;1* mutant *Arabidopsis* with *AtHKT1;1pro::SvHKT1;1* construct. Transcripts of *AtHKT1;1* or *SvHKT1;1* were detected in WT, mutant, and two independent lines of transformed *Arabidopsis* plants (three biological replicates) by RT-PCR (**A**). The appearance (**B**) and fresh weight (**C**) of the plants grown for two weeks on 100 mM NaCl medium. Na^+^ (**D**,**F**) and K^+^ (**E**,**G**) concentrations in the shoots (**D**,**E**) and roots (**F**,**G**) of the plants. Data are presented as means ± SD (*n* = 9 (**B**,**C**) and *n* = 3 (**D**–**G**)). Please note that each panel has a different *Y*-axis scale. Single and double asterisks denote significant differences compared with the values of WT plants of the same conditions at *p* < 0.05 and *p* < 0.01, respectively, determined using the Student’s *t*-test.

We transformed *athkt1;1 Arabidopsis* plants in which the gene for *AtHKT1;1* sodium transporter was tagged with T-DNA. The *AtHKT1;1* transcript was not detected in the mutant line, and the *SvHKT1;1* transcript was detected in two independent transgenic mutant lines (*athkt1;1/SvHKT1;1-B* and *-C*) transformed with the *SvHKT1;1* gene driven by the *AtHKT1;1* promoter by semi qRT-PCR (Figure 5A). When transferred onto 100 mM NaCl medium, the transgenic lines, as well as *athkt1;1* mutant line, showed diminished growth (Figure 5B,C). This result indicated that *SvHKT1;1* does not complement the defected AtHKT1;1 function. Root Na^+^ concentrations in both the mutant and the transgenic lines were significantly increased compared to that in WT plants, despite the loss of functionality of *AtHKT1;1*, whereas their shoot Na^+^ concentrations increased, but not significantly, under salt stress (Figure 5D,F). Interestingly, root K^+^ concentrations in the transgenic lines decreased significantly or tended to decrease compared to that in WT plants, although no difference was observed among their shoot K^+^ concentrations (Figure 5E,G).

### 2.7. Constitutive Expression of SvHKT1;1 in Wild-Type Arabidopsis Plants

**Figure 6:**
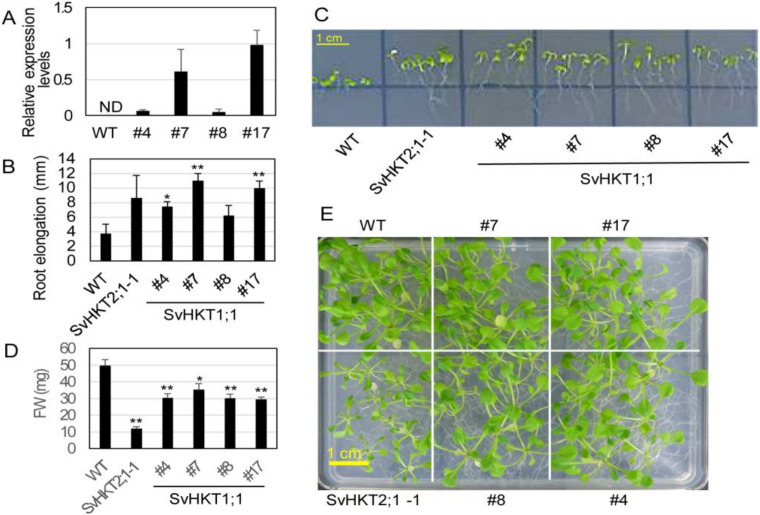
Expression level of the transgene, root growth, and salt tolerance of transgenic *Arabidopsis* plants expressing *SvHKT1;1*. (**A**) Expression levels of transgenes in the SvHKT1;1 transgenic lines. *Actin* was used as an internal standard. ND; not detected. Data are presented as means ± SE (*n* = 3 biological replicates). (**B**) Root elongation of transgenic and WT seedlings grown on 0.1 mM K^+^ medium. Data are presented as means ± SE (*n* = 3 biological replicates). (**C**) The appearance of transgenic lines and WT seedlings on 0.1 mM K^+^ medium examined in panel B. (**D**) Fresh weight (FW) of WT and the transgenic lines. One-week-old seedlings germinated on 1/2 MS agar medium were transplanted onto 1/2 MS agar medium supplemented with 50 mM NaCl, and their FW was determined after another two weeks of incubation. Data are presented as means ± SE (*n* = 10 biological replicates). (**E**) The appearance of plants examined in panel D. Single and double asterisks denote significant differences compared with the values of WT plants of the same conditions at *p* < 0.05 and *p* < 0.01, respectively, determined using the Student’s *t*-test. Single and double asterisks denote significant differences compared with the values of WT plants of the same conditions at *p* < 0.05 and *p* < 0.01, respectively, determined using the Student’s *t*-test.

We introduced *35S::SvHKT1;1* into WT *Arabidopsis* and examined the expression levels of the transgenes in four T_2_ lines with putative single transgenes (#4, 7, 8, and 17), judging from the segregation ratio of the hygromycin tolerant T_3_. There was a large variation in the expression level (Figure 6A). We examined root growth of the transgenic lines on 0.1 mM K^+^ medium, because expression of K^+^/Na^+^ symporters, *SvHKT2;1* and *SvHKT2;2*, in *Arabidopsis* resulted in enhanced root growth under K^+^-starved conditions in our previous study [16]. The SvHKT1;1 transgenic lines showed elongated root growth compared with WT plants, while the root growth of WT plants was severely inhibited (Figure 6B,C). A weak correlation was observed between the values of root elongation and the transcription levels.

Then, we assessed salt tolerance of the SvHKT1;1 lines at seedling stage in comparison with WT plants and the SvHKT2;1-1 line, which showed reduced salt tolerance in our previous study [40]. *Arabidopsis* seedlings were transferred onto 1/2 MS agar medium supplemented with 50 mM NaCl, incubated for a further 14 days, and their shoot fresh weight (FW) was determined. SvHKT1;1 lines showed diminished growth on 50 mM NaCl medium, although not as severely as that of the SvHKT2;1-1 line (Figure 6D,E). These results indicated that constitutive expression of *SvHKT1;1* increased salt sensitivity of the transgenic lines.

**Figure 7:**
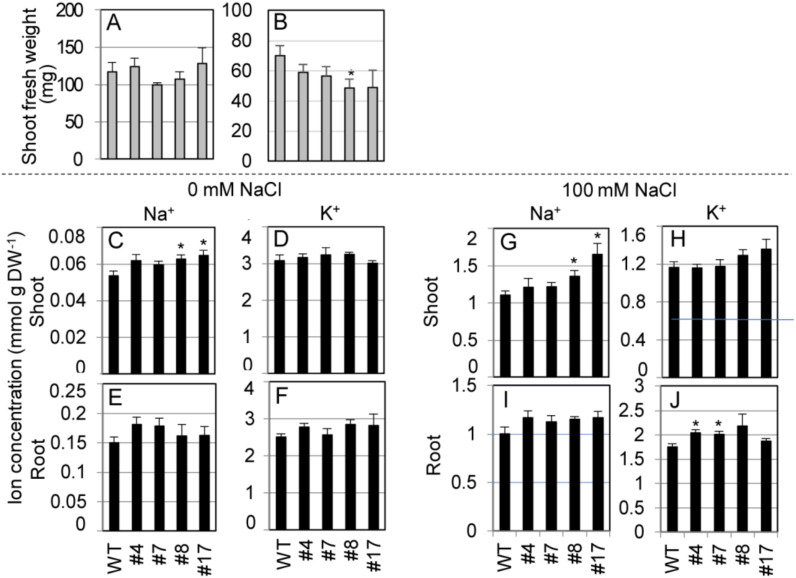
Shoot fresh weight and ion concentrations of *SvHKT1;1* transgenic lines and WT plants. (**A**,**B**) Shoot fresh weight of WT plants and the transgenic lines. Two-week-old seedlings germinated on 1/2 MS agar medium were hydroponically cultured in 1/2 MS liquid medium for another one week, and then cultured in 1/2 MS medium supplemented with 0 (**A**) or 100 mM (**B**) NaCl, and their FW was determined after one week. (**C**–**J**) Ion concentrations in WT plants and the transgenic lines. Na^+^ (**C**,**E**,**G**,**I**) and K^+^ (**D**,**F**,**H**,**J**) concentrations in their shoots (**C**,**D**,**G**,**H**) and roots (**E**,**F**,**I**,**J**) were determined. Data are presented as means ± SE (*n* = 3–4 biological replicates). Please note that each panel has a different *Y*-axis scale. Single and double asterisks denote significant differences compared with the values of WT plants of the same conditions at *p* < 0.05 and *p* < 0.01, respectively, determined using the Student’s *t*-test.

We measured shoot FW and ion concentrations in shoots and roots of the transgenic lines hydroponically cultured in 1/2 MS liquid medium supplemented with 0 or 100 mM NaCl at the bolting stage (Figure 7). Under nonstress conditions (0 mM NaCl), there was no difference between shoot growth of the transgenic lines and WT plants (Figure 7A). Under salt stress, the transgenic lines showed significantly or relatively smaller growth than WT plants (Figure 7B). Under nonstress condition, shoot Na^+^ concentrations in two transgenic lines were significantly higher and the other two were higher than those in WT plants, while no significant differences were observed in shoot K^+^ between SvHKT1;1 lines and WT plants (Figure 7C,D). No significant differences were observed in their root Na^+^ and K^+^ concentrations (Figure 7E,F). Under salt stress, shoot Na^+^ and root K^+^ concentrations in the transgenic lines were significantly or relatively higher than those of WT plants (Figure 7G,J). No significant differences were observed in shoot K^+^ and root Na^+^ concentrations between SvHKT1;1 lines and WT plants (Figure 7H,I).

### 2.8. K^+^ and Na^+^ Concentrations in the Xylem and Phloem Saps of Arabidopsis Expressing SvHKT1;1

**Figure 8:**
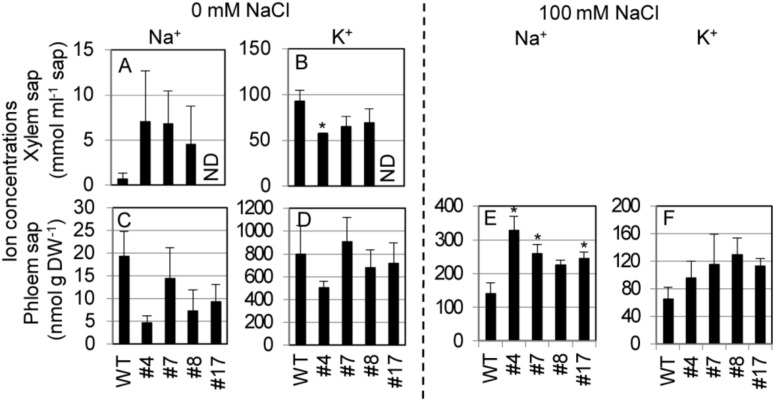
Na^+^ and K^+^ concentrations in the xylem and phloem saps from *SvHKT1;1* transgenic lines and WT plants. (**A**–**D**) Ion concentrations in the xylem and phloem saps of WT plants and the transgenic lines under nonstress condition. Plants were hydroponically cultured in 1/2 Hoagland liquid solution until the bolting stage, and Na^+^ (**A**,**C**) and K^+^ (**B**,**D**) concentrations in their xylem (**A**,**B**) and phloem (**C**,**D**) saps were determined. (**E**,**F**) Ion concentrations in the phloem saps of WT plants and the transgenic lines under 100 mM NaCl. ND; not determined. Three-week-old plants were subjected to 1/2 Hoagland liquid solution supplemented with 100 mM NaCl for seven days, and Na^+^ (**E**) and K^+^ (**F**) concentrations in their phloem saps were determined. Xylem saps were not obtained from salt-treated plants. Data are presented as means ± SE (*n* = 3–4 biological replicates). Please note that each panel has a different *Y*-axis scale. Single and double asterisks denote significant differences compared with the values of WT plants of the same conditions at *p* < 0.05 and *p* < 0.01, respectively, determined using the Student’s *t*-test.

To examine the mode of SvHKT1;1-mediated K^+^ and Na^+^ transport in *Arabidopsis* plants, *SvHKT1;1* lines and WT plants were hydroponically cultured in 1/2 Hoagland solution, and K^+^ and Na^+^ concentrations in the xylem and phloem saps were determined at the bolting stage (Figure 8). We used Hoagland solution in this experiment because *Arabidopsis* plants showed better growth performance in 1/2 Hoagland solution than in 1/2 MS medium. Under nonstress condition, Na^+^ concentrations in the xylem saps of SvHKT1;1 lines were higher, but not significantly, than those in WT plants (Figure 8A), and Na^+^ concentrations in the phloem saps were lower, but not significantly, in SvHKT1;1 lines than those in WT plants (Figure 8C). K^+^ concentrations in the xylem and phloem saps were similar among the transgenic lines and WT plants, except for xylem sap of the #7 line (Figure 8B,D). In contrast, the phloem sap Na^+^ concentrations were significantly higher in SvHKT1;1 lines than in WT plants under 100 mM NaCl conditions (Figure 8E). The phloem sap K^+^ concentrations were similar among the transgenic lines and WT plants (Figure 8F). We could not obtain xylem sap from the transgenic lines and WT plants under salt stress.

### 2.9. Ion Uptake and Translocation Rates in Arabidopsis Seedlings after Salt Treatment

**Figure 9:**
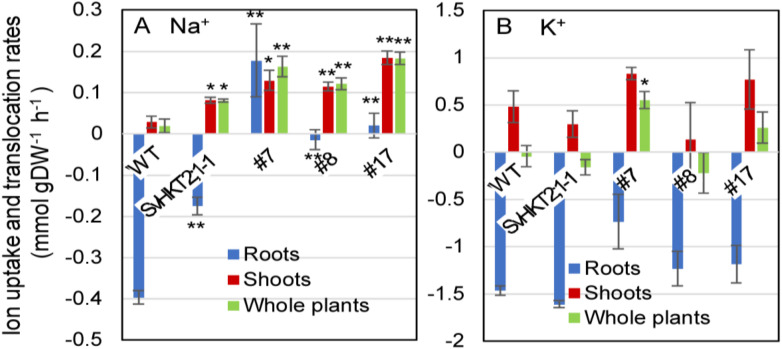
Na^+^ and K^+^ uptake and translocation rates in *SvHKT1;1* transgenic lines and WT plants under salt stress. Twelve-day-old *Arabidopsis* seedlings, which were pre-incubated in 1/2 MS liquid medium for 24 h to adapt to liquid medium, were transferred into micro cuvettes filled with 1/2 MS liquid medium supplemented with 100 mM NaCl and further incubated for one hour. Their roots and shoots were separately harvested before and after the treatment, dried overnight, and weighted. Na^+^ (**A**) and K^+^ (**B**) uptake and translocation rates in the roots, shoots, and whole plants under salt stress were calculated and expressed as mmol per g of dry weight per hour of salt treatment (mmol·g DW^–1^·h^–1^). Ten seedlings were pooled and used as one sample. Data are presented as means ± SE (*n* = 3 biological replicates). Single and double asterisks denote significant differences compared with the values of WT plants at *p* < 0.05 and *p* < 0.01, respectively, determined using the Student’s *t*-test.

To examine the mode of Na^+^ and K^+^ uptake, release, or translocation by SvHKT1;1 in the roots of transgenic lines, we subjected the transgenic and WT seedlings to liquid 1/2 MS medium supplemented with 100 mM NaCl for 1 h and measured the changes of their Na^+^ and K^+^ concentrations at the early stage of saline stress (Figure 9). While Na^+^ uptake rate in roots of WT plants and SvHKT2:1-1 line took negative values, indicating their Na^+^ release, SvHKT1;1 lines showed significantly increased or almost unchanged uptake rate after they were transferred to 100 mM NaCl medium (Figure 9A). Na^+^ translocation rates in both shoots and whole plants of all transgenic lines were significantly higher than those in WT plants (Figure 9A). These results indicated enhanced Na^+^ uptake and translocation rates in the transgenic lines compared with WT plants. There were no differences in K^+^ uptake and translocation rates among the transgenic lines and WT plants, except for whole plants of SvHKT1;1 transgenic line #7 (Figure 9B).

## 3. Discussion

We isolated a gene for class I *HKT*, *SvHKT1;1*, from a halophyte *S. virginicus*. The deduced AA sequence contains a serine in the first P-loop, which is common to HKT1 sodium transporters [37,38]. Electrophysiological analysis showed that SvHKT1;1 mediates inward and outward Na^+^, but not K^+^, transport in *X. laevis* oocytes (Figure 4), which was commonly observed in typical glycophytic HKT1s. SvHKT1;1 did not complement K^+^ transport activity in K^+^ transporter-defective mutant yeast. Thus, SvHKT1;1 was proven to be a typical Na^+^ monoporter.

Although HKT1s were reported to have diverse expression patterns in both dicotyledonous and monocotyledonous plants, the expression profile of *SvHKT1;1* is unique compared with that of other *HKT1s.* The transcript was abundant in the shoots compared with the roots and was upregulated by severe salt stress (500 mM NaCl), but not by mild or moderate salt stress (less than 300 mM) (Figure 2). *AtHKT1;1* expression was reported to be slightly induced by mild salt stress [20,41,42]. *OsHKT1;1* expression in rice was associated with the phloem and xylem of leaves and roots, and its transcripts were induced in shoot but not in roots [23,25], while the induction of *OsHKT1;5* expression by salt stress was found in the roots but not in shoots [24,26]. *OsHKT1;4* transcripts were prominent in leaf sheaths and stem [27]. In wheat, an *OsHKT1;5*-like gene, *TmHKT1;5-A*, showed root-specific constitutive expression and was not induced by NaCl [31]. On the other hand, expression of *EsHKT1;2* and *EpHKT1;2* in halophytic *Arabidopsis* relatives was dramatically induced by salt stress (150 mM) [33,43]; however, EsHKT1;2 and EpHKT1;2 showed K^+^ uptake ability and were, therefore, functionally distinguished from SvHKT1;1. Thus, each *HKT1* has a diverse expression profile, and their expression patterns may reflect the unique Na^+^ management strategy of each plant.

Downregulation of *EsHKT1;2* in *E. salsuginea* leads to a hyper-salt-sensitive phenotype under K^+^-deficient conditions [33,35], and overexpression of *EpHKT1;2* enhanced its salt stress tolerance [43]. Based on these findings, these genes could be considered major contributors to the halophytic nature of *E. salsuginea* and *E. parvula* [43]. It was pointed out that HKT1s in non-halophytes are also associated with salt tolerance. Na^+^ removal from root xylem sap and/or shoot phloem was reported for HKT1s including AtHKT1;1 in *Arabidopsis* [19,20,21,22], OsHKT1;1 and OsHKT1;5 in rice [23,24,25], TaHKT1;5-D in bread wheat [28], HvHKT1;1 and HvHKT1;5 in barley [29,30], and TmHKT1;5-A in wheat [31]. Phylogenetic analysis showed that SvHKT1;1 is evolutionally close to some of these HKT1s, such as OsHKT1;1 and HvHKT1;1 (Figure 1); however, *SvHKT1;1* driven by the *AtHKT1;1* promoter did not complement the *athkt1;1 Arabidopsis* mutant (Figure 6), indicating the distinguished function of SvHKT1;1 from these HKT1s.

The shoot and root Na^+^ concentrations in *S. virginicus* increased linearly in accordance with the NaCl concentration in culture solution up to 300 mM NaCl, where *SvHKT1;1* was not upregulated, but the Na^+^ concentration decreased at 500 mM, where *SvHKT1;1* was dramatically upregulated (Figure 2). This well-synchronized pattern between the gene expression and the decrease in shoot Na^+^ concentration suggested that SvHKT1;1 could be involved in Na^+^ excretion from shoots in *S. virginicus* and is upregulated only when the plants need to cope with extremely severe salinity. This mechanism may make it possible for *S. virginicus* to accumulate shoot Na^+^ under salinity stress but not to exceed levels required for osmotic adjustment. It was suggested that a plant’s ability to exclude Na^+^ is positively correlated with the overall salinity tolerance in glycophytes, including wheat, sorghum, maize, and tomato [31,44,45,46,47,48]. However, the essentiality of Na^+^ exclusion may differ depending on severity of the stress [49] and the capacity of Na^+^ sequestration in the shoot (tissue tolerance). Comparison of barley cultivars of different salt tolerance suggested that plants need to rapidly adjust their shoot osmotic potential by sending an appropriate amount of Na^+^ to the shoot within the first few days and shutting down any further Na^+^ delivery to the shoot [49]. We reported that *S. virginicus* also gradually accumulates a certain amount of Na^+^ in shoots under 500 mM NaCl conditions over five days, but regulates so that the Na concentration does not excess a certain level [17]. A halophytic relative of *A. thaliana*, *T. halophila* (*E. salsuginea*), accumulated less Na^+^ and more K^+^ than *A. thaliana* during short-term (25 h) exposure to salt stress; however, after long-term exposure (5 weeks), *T. halophila* accumulated more Na^+^ than *A. thaliana* [50]. Thus, halophytes have the property that they do not accumulate high concentrations of Na^+^ in the short term. The expression of *SvHKT1;1* in *S. virginicus* was dramatically upregulated at 6 h after 500 mM NaCl treatment but not under 300 mM NaCl (Figure 2). This early response of *SvHKT1;1* in high Na^+^ conditions may be responsible for the Na^+^ accumulation in the short term. Therefore, we hypothesize that SvHKT1;1 could play a major role in preventing excess Na^+^ accumulation in *S. virginicus* shoots under high-saline conditions. To test this hypothesis, more detailed spatial–temporal expression profiling of SvHKT1;1 and loss-of-function experiments in *S. virginicus* are needed.

In this study, we investigated the function of SvHKT1;1 in transgenic *Arabidopsis* because our attempt to transform *S. virginicus* was unsuccessful and, thus, the knockout or knockdown line is not available. The transgenic lines showed elongated root growth on low-K^+^ medium (containing 0.1 mM K^+^ and 0.725 mM /Na^+^), where root growth of WT plants is severely inhibited (Figure 6B,C). A similar phenotype was observed in *Arabidopsis* expressing K^+^/Na^+^ symporters, SvHKT2s [16]. These results indicated that enhanced root growth of the transformants under low K^+^ conditions was due to an increase in the ability to absorb Na^+^ but not K^+^.

Overexpression of *AtHKT1;1* specifically in the root xylem parenchyma cells of *Arabidopsis* improved Na^+^ exclusion and salinity tolerance [51], and root cortical and epidermal cell-specific expression of *AtHKT1;1* in rice enhanced salinity tolerance [52]. These results indicate that the major role of AtHKT1;1 in salt tolerance is Na^+^ exclusion from root xylem. However, surprisingly, constitutive overexpression of *AtHKT1* in potato also reduced Na^+^ accumulation in leaves and enhanced salt tolerance [53]. In this study, shoot Na^+^ concentrations in transgenic *Arabidopsis* lines were significantly higher than those of WT plants under 100 mM NaCl conditions (Figure 7G). Na^+^ concentrations in xylem saps were relatively higher than those in WT plants under nonstress conditions (Figure 8A). Measurement of Na^+^ uptake and translocation rates also indicated enhanced Na^+^ uptake in roots of *SvHKT1;1* lines under salinity conditions (Figure 9A). Considering these results together, it was suggested that constitutive expression of *SvHKT1;1* enhanced Na^+^ uptake in root epidermal cells and then increased Na^+^ transport to shoots, which led to reduced salt tolerance (Figure 5 and Figure 7). On the other hand, Na^+^ concentrations in phloem sap of the SvHKT1;1 lines were not significantly different from those in WT plants under nonstress conditions (Figure 8C); however, the transgenic lines showed higher phloem sap Na^+^ concentrations than WT plants under 100 mM NaCl conditions (Figure 8E). These results may suggest that SvHKT1;1 mediated Na^+^ uploading into phloem when an excess amount of Na^+^ was accumulated in shoots to translocate Na^+^ to roots. Since shoot Na^+^ concentration in the transgenic lines under 100 mM NaCl (Figure 7H) was six times higher than that in *S. virginicus* under 300 mM NaCl (Figure 2F), the condition may be adequate for SvHKT1;1 to mediate Na^+^ uploading to phloem. These data further support our hypothesis that SvHKT1;1 could play a major role in preventing excess Na^+^ accumulation in *S. virginicus* shoots under high-saline conditions, although tissue specificity of *SvHKT1;1* expression was not revealed.

## 4. Materials and Methods

### 4.1. Isolation of SvHKT1;1 Gene

We searched for HKT gene homologs in previously constructed unigenes assembled from *S. virginicus* RNA-Seq data [36], and we found HKT-like unigenes. Among them, one unigene sequence, which is similar to sodium transporter *AtHKT1;1* gene, *SvHKT1;1* (DDBJ (DNA Data Bank of Japan). accession number LC545616), was PCR-amplified using specific primers, SvHKT1B1F 5′–CACCATGCATCCAGCCAGTTCAGTTCTA–3′ and SvHKT1B2R 5′–TCCTTGAGGTCATGGAGTTGG–3′. Amplified sequences were cloned into pENTER vectors (Thermo Fisher Scientific, Tokyo, Japan) to form the entry vector, pENTER-SvHKT1;1.

### 4.2. Phylogenetic Analysis

A phylogenetic analysis of the HKT amino-acid (AA) sequences using the neighbor-joining method, following their alignment using ClustalW, was performed using the MEGA-X software package [39]. Accession numbers for amino-acid sequences of HKTs used for phylogenetic analysis are listed in Supplementary Figure S1.

### 4.3. Real-Time qRT-PCR

*S. virginicus* plants were hydroponically cultivated in 1/2 MS salt solution, and then transplanted to 1/2 MS salt solution supplemented with 0, 100, 300, or 500 mM NaCl treatments. Shoots and roots (*n* = 3 biological replicates) were separately harvested for RNA isolation and ion measurement 48 h after the treatments. The RNAiso plus (TakaraBio, Ohotsu, Japan) was used to extract the total RNA, and real-time qRT-PCR was performed as previously reported [36]. A pair of primer sets, qSvHKT1BF 5′–CTTGGCCCACATAGTATCAGG–3′ and qSvHKT1BR 5′–GGTGAAGATGGAGAAGGTGCATAC–3′, was used. The relative expression levels of the target to reference genes, *eukaryotic translation initiation factor 3 subunit-like protein* (*eIF3*) and *actin* from *S. virginicus*, was detected using primer sets, qSveIF1F 5′–ACATGTGAGTCTGACCTCGTCGAC–3′ and qSveIF2R 5′–TGAGCAAGCCAATGGCCTTCTCAG–3′ and SvActinF 5′–CAGATCATGTTCGAGACCTTC–3′ and SvActinR 5′–GACGGTGTGGCTGACACCAT–3′, respectively, and they were calculated using the delta-delta Ct method.

Similarly, RNA was extracted from 14-day-old *Arabidopsis* plants grown on 1/2 MS medium. Real-time qRT-PCR analysis was performed as previously reported [16] using primer sets for *SvHKT1;1* and *ubiquitin extension protein 5* (*UBQ5*), UBQ5F 5′–TGTGAAGGCGAAGATCCAAG–3′, and UBQ5R 5′–GAGACGGAGGACGAGATGAAG–3′ as a reference.

For semi-quantitative RT-PCR analysis, first-strand complementary DNA (cDNA) was synthesized from 250 ng of total RNA using a QuantiTect Reverse Transcription Kit (Qiagen, Tokyo, Japan) according to the manufacturer’s instructions and diluted 20-fold, and 1 µL was used as a template. Semi qRT-PCR was carried out for 30 cycles of 98 °C for 10 s, followed by 60 °C for 10 s, and 68 °C for 60 s, using Tks Gflex DNA Polymerase (TakaraBio). In addition to primer pairs for *SvHKT1;1* and *UBQ5*, primer sets for *AtHKT1;1*, AtHKT101F 5′–GAGAACTAAAATGGACAGAGTGGTG–3′ and AtHKT102R 5′–GTACCAAGATAGCTGGGGAAAGTG–3′, were used.

### 4.4. Subcellular Localization of SvHKT1;1 in Nicotiana benthamiana Leaves

To examine the subcellular localization of SvHKT1;1, the entry vector pENTER-SvHKT1;1 was reacted with a destination vector, pH7WGF2.0, encoding an N-terminal EGFP fusion [54] using LR clonase reactions (Thermo Fisher Scientific). As a control, a nonfused EGFP construct was used. The recombinant plasmids were transformed into *Agrobacterium tumefaciens* strain GV3101, and then infiltrated into *N. benthamiana* leaves. Two days post infiltration, GFP fluorescence and differential interference contrast images were observed using an FX3000 confocal fluorescence microscope (Olympus, Tokyo, Japan). To observe plasmolyzed cells, leaf cells were treated with 500 mM mannitol for 30 min.

### 4.5. Functional Analysis of SvHKT1;1 in X. laevis Oocytes

The *SvHKT1;1* cDNA was PCR-amplified using primers to produce the entry vector pENTER-SvHKT1;1, excised from the entry vectors using the restriction enzymes *Not*I and *Asc*I, and then inserted into the *Not*I and *Asc*I sites of pXBG-NA [16]. A mMESSAGE mMACHINE in vitro transcription kit (Thermo Fisher Scientific) was used to synthesize the capped-analogue RN. Oocytes and TEVC experiments were prepared and performed as described previously [16], with a minor modification. In brief, 12.5 ng of cRNA of *SvHKT1;1* was injected into *X. laevis* oocytes and incubated at 18 °C for two days. Water-injected oocytes were also prepared as negative controls. The data recordings and analysis were performed using an Axoclamp 900 A amplifier and an Axon Instruments Digidata 1440 A with Clampex 10.3 and Clampfit 10.3 software (Molecular Devices, Sunnyvale, CA, USA). The analyses of ion selectivity using alkali cation salts used oocytes bathed in a background solution containing 96 mM NaCl, KCl, or Na-glutamate salts, adjusted to pH 7.5. The background solution also contained 1.8 mM CaCl_2_, 1 mM MgCl_2_, 1.8 mM mannitol, and 10 mM 4- (2-hydroxyethyl)-1-piperazineethanesulfonic acid (HEPES) for NaCl or KCl salt, and 1.8 mM Ca-Glu, 1 mM Mg-Glu, 1.8 mM mannitol, and 10 mM HEPES for Na-Glu salt. The experiments using frog oocytes were approved by the Animal Care and Use Committee, Okayama University (approval number OKU-2017271 on 26 June 2017) that follows the related international and domestic regulations.

### 4.6. Production of Transgenic Arabidopsis

The entry vector pENTR-SvHKT1;1 was reacted using LR enzyme (Thermo Fisher Scientific) with a destination vector pGH1 [16] and pAtHKT1 [42], to form pGH1-SvHKT1;1 and pAtHKT1-SvHKT1;1, in which the transgenes are driven by *CaMV35S* and *Arabidopsis AtHKT1;1* promoters, respectively. *Arabidopsis* wild-type (WT) plants (ecotype Columbia) and a T-DNA-tagged *athkt1;1* mutant line (ABRC Stock Number: CS372002 [55]) were transformed with expression vectors pGH1-SvHKT1;1 and pAtHKT1-SvHKT1;1, respectively, by floral dipping [56]. *Agrobacterium* strain GV3101 was used for transformation.

### 4.7. Cultivation of Arabidopsis Plants

Seeds of *Arabidopsis* were sown on 1/2 MS agar medium (1/2 MS salts, 1% sucrose and 0.8% agar at pH 5.7). The plants were grown at 23 °C under a 16-h/8-h light/dark cycle with approximately 60 µmoL·m^−2^·s^−1^ light intensity. For salt stress treatment at seedling stage, seven-day-old seedlings were transplanted onto 1/2 MS agar medium supplemented with 50 mM NaCl and incubated for a further 14 days.

Hydroponic culture of transgenic *Arabidopsis* was performed using the Home Hyponica Karen (Kyowa Co., LTD, Osaka, Japan) system with 1/2 MS medium or 1/2 Hoagland salt solution [57] supplemented with 0.2% 2-(N-morpholino)ethanesulfonic acid (MES) as the hydroponic culture solution. Fourteen-day-old plants grown on 1/2 MS agar medium were transplanted to the hydroponic system. For salt stress treatment at bolting stage, the hydroponic culture solution was replaced with 1/2 MS medium supplemented with 0 or 100 mM NaCl and 0.2% MES at the age of 24 days, when plants had almost started bolting. For RNA extraction, 14-day-old *Arabidopsis* plants cultivated on 1/2 MS agar medium were used.

### 4.8. Measurement of Ion Concentrations in Plants

Measurement of ion concentrations in plants was performed as described previously using an Ion Analyzer IA-300 (Toa DKK, Tokyo, Japan) [16].

### 4.9. Collection of Xylem and Phloem Saps

Transgenic and WT *Arabidopsis* plants were grown on 1/2 MS plate medium supplemented with 1% sucrose (pH 5.7) for two weeks, and then were transplanted to liquid 1/2 Hoagland solution supplemented with 0.2% MES (pH 5.7) until they reached the bolting stage. Collection of xylem and phloem sap was carried out according to the methods of Sunarpi et al. [20]. The collected samples were used for ion measurement using an Ion Analyzer IA-300.

### 4.10. Measurement of Ion Uptake and Translocation Rates in Arabidopsis Seedlings after Salt Treatment

Rates of Na^+^ and K^+^ uptake or release in WT plants and the transgenic lines were determined based on the changes in their Na^+^ and K^+^ concentrations after exposure to salinity stress. Seedlings at 12 days old grown on 1/2 MS agar medium supplemented with 1% sucrose were used. Ten seedlings were pooled and used as one sample. Nine pooled samples were prepared for each line. The pooled samples were transferred into microcuvettes filled with 3.0 mL of 1/2 MS liquid medium, taking care to fully immerse the roots into the medium; finally they were incubated at 23 °C under approximately 60 µmoL·m^−2^·s^−1^ light intensity. After 24 h of incubation, three pooled samples were harvested from each line as samples before salt treatment (zero-time samples). They were briefly washed with pure water, harvested by dividing into shoots and roots, and dried at 60 °C overnight to determine the dry weight (DW). The remaining pooled samples were briefly washed in liquid 1/2 MS medium supplemented with 100 or 200 mM NaCl (three pooled samples for each condition), transferred to another microcuvette filled with 3.0 mL of 1/2 MS medium supplemented with 100 or 200 mM NaCl. After 60 min of incubation, seedlings were briefly washed in pure water, harvested as salt-treated samples (samples after 60 min) by dividing into roots and shoots, and dried at 60 °C overnight to determine their DW. The change in Na^+^ and K^+^ concentrations in the roots, shoots, and whole plants by 60 min of 100 or 200 mM NaCl treatment was calculated and expressed as millimoles per gram of dry weight per hour (mmol·g DW^–1^·h^–1^).

## 5. Conclusions

SvHKT1;1 from a halophytic turf grass, *S. virginicus*. SvHKT1;1 is an Na^+^ transporter and its expression is abundant in the shoots compared with the roots in *S. virginicus* and interestingly upregulated only by severe salt stress (500 mM NaCl). Arabidopsis constitutively expressing *SvHKT1;1* showed higher shoot Na^+^ concentrations and lower salt tolerance than WT plants by its enhanced Na^+^ uptake in roots. Na^+^ concentrations in phloem sap of the transgenic Arabidopsis were higher than those in WT plants under salt stress These results suggested possibility that SvHKT1;1 plays a role in preventing excess shoot Na^+^ accumulation in *S. virginicus*.

## Supplementary Materials

Supplementary materials can be found at https://www.mdpi.com/1422-0067/21/17/6100/s1: Figure S1: Alignment of partial amino-acid sequences of selected class I HKTs, Figure S2: Expression profiles of SvHKT1;1 gene in *Sporobolus virginicus*, Figure S3: Growth of yeast strain 9.3 transformed with the empty vector or the plasmid containing SvHKT2;1 or SvHKT1;1 gene, Table S1: Accession numbers for amino-acid sequences of HKTs used for phylogenetic analysis.
