# Supplementary material for: Na+ Transporter SvHKT1;1 from a Halophytic Turf Grass Is Specifically Upregulated by High Na+ Concentration and Regulates Shoot Na+ Concentration
Source: Int J Mol Sci. 2020 Aug 24;21(17):6100. doi: 10.3390/ijms21176100 (PMC7503356; doi:10.3390/ijms21176100)
Supplement: Supplementary file 1 [file ijms-21-06100-s001.zip › Table S1.pdf]

Supplemental Table 1. Accession numbers for amino acid sequences of HKTs used for phylogenetic analysis

HKT	Accession numbers
SvHKT1;1	LC545616
SvHKT2;1	LC271218
SvHKT2;2	LC271219.1
AtHKT1;1	AK228564.1
OsHKT1;1	XP_015641242.1
OsHKT1;3	XP_015626193.1
OsHKT1;4	XP_015633970.1
OsHKT1;5	XP_015631953.1
HvHKT1;1	JF496205.1
HvHKT1;5	ABK58096.1
GmHKT1A	KRH25841.1
GmHKT1B	KRH55676.1
GmHKT1C	KRH74148.1
VaHKT1B	NC_028351.1
SlHKT1;1	HE962483.1
SlHKT1;2	HE962484.1
McHKT1	AAK52962.1
McHKT1;2	AAO73474.1
PtrHKT1;1	XP_002325229.1
EcHKT1;1	AF176035.1
EcHKT1;2	AF176036.1
SsHKT1	AAS20529.2
MtHKT1;5	AES77170.2
Ts(Es)HKT1;1	JQ063120.1
Ts(Es)HKT1;2	BAJ34563.1
CaHKT1;1	AFH37929.1
SiHKT1;1	XM_014805535.2
TaHKT1;5-D (TaHKT8)	ABG33949.1
TaHKT8B	ABG33947.1
TmHKT1;5-A (TmHKT8)	ABG33946.1
SbHKT6	XP_002451638.1
ZmHKT1	AEK27028.1
SbHKT1	XM_002438915.2
SbHKT7	XP_002448492.2
SpHKt1;1	Tp6g07120*
SpHKT1;2	Tp6g07110*
BdHKT1	XP_003560515.1
BdHKT6	XP_003570995.1
TaHKT2;1	AAA52749.1
HvHKT2;1	AEM55590.1
OsHKT2;1	BAB61789.1

* http://thellungiella.org/blast/db/TpV84ORFs.protein
