# Supplementary material for: Na+ Transporter SvHKT1;1 from a Halophytic Turf Grass Is Specifically Upregulated by High Na+ Concentration and Regulates Shoot Na+ Concentration
Source: Int J Mol Sci. 2020 Aug 24;21(17):6100. doi: 10.3390/ijms21176100 (PMC7503356; doi:10.3390/ijms21176100)
Supplement: Supplementary file 1 [file ijms-21-06100-s001.zip › Supplemental Figs.pdf]

```
AtHKT1;1  30:LFFSFLGFLALKITKPRTTSRPHDFDLFFTSVSAITVSSMSTVDMEVFSN:79
HvHKT1;1  91:LSISFAGFLALKNLRPLNKPSPRNLDLMFTSVSTVTVSSMATVEMEEFSG:140
OsHKT1;1  81:VIISFAGFLALKNLKPQGKPGPKDLDLLFTSVSTLTVSSMATVEMEDLSD:130
SiHKT1;1  81:MAISFAGFVALKNLNPHGKPVPSDFDLMFTSVSTATVSSMSTIQMEDLSD:130
SvHKT1;1  81:MVTSFVGFLALKNLQSLGKPVLRDLDLMFTSVSTVTVSSMSTVQMEDLSE:130
```

P_A

Fig. S1. Alignment of partial amino acid sequences of selected class I HKTs. Amino acid sequences of AtHKT1;1 (AK228564.1), HvHKT1;1 (JF496205.1), OsHKT1;1 (XP_015641242.1), SitHKT1;1 (XM_014805535.2), and SvHKT1;1 (LC545616) were aligned. Conserved serine residues among class I HKT sequences are surrounded by a square. The pore A loop region (P_A) is colored pink.

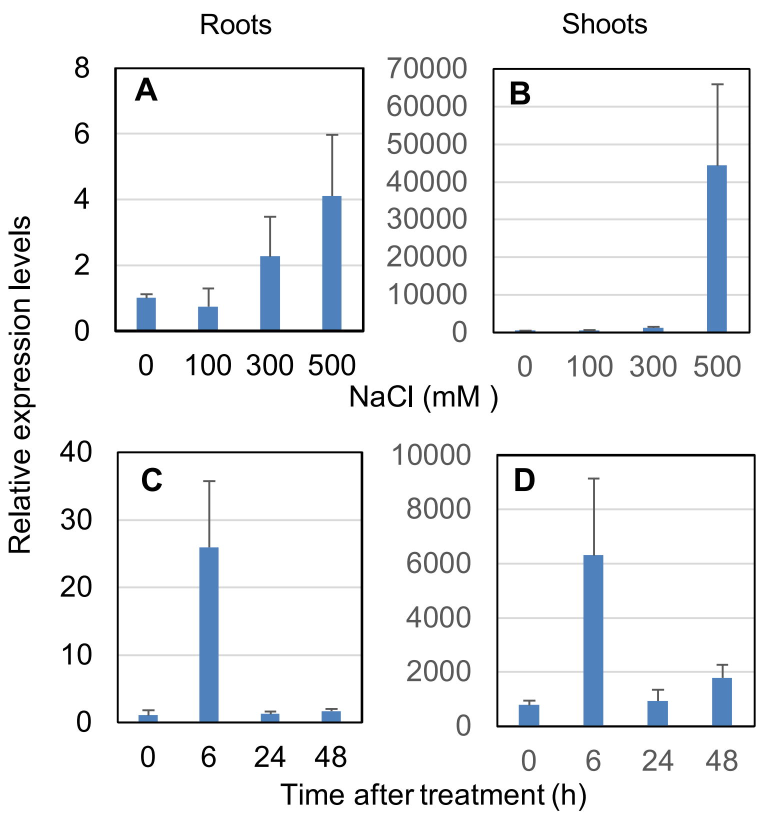

Fig. S2. Expression profiles of *SvHKT1;1* gene in *Sporobolus virginicus.*
The expression profiles of *SvHKT1;1* gene in roots and shoots of hydroponically grown *S. virginicus* were determined using qRT-PCR under different salt stress conditions (A, B) or at different time points after salt treatment (C, D). Plants grown in 1/2 MS medium were transferred to 1/2 MS medium supplemented with 0, 100, 300, or 500 mM NaCl and the roots (A) and shoots (B) were harvested at 48 h after the treatment. Plants grown in 1/2 MS medium were transferred to 1/2 MS medium supplemented with 500 mM NaCl and the roots (C) and shoots (D) were harvested at indicated time points. Expression levels relative to that in roots at 0 h after treatment (1.0) are shown. The *Actin* was used as a reference gene. Data are presented as mean ± SE (n = 3, biological replicates).

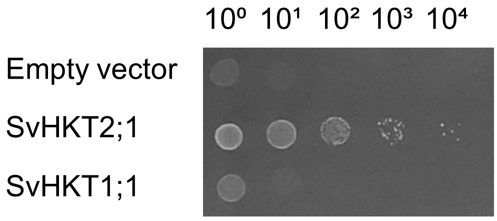

Fig. S3. Growth of yeast strain 9.3 transformed with the empty vector or the plasmid containing *SvHKT2;1* or *SvHKT1;1* gene.

SC/-His medium supplemented with 0.2 mM K^+ (0.7 mM Na^+) were inoculated with serially diluted yeast cell suspensions and incubated for 3 d.
